# Mobile Phone Middleware Architecture for Energy and Context Awareness in Location-Based Services

**DOI:** 10.3390/s141223673

**Published:** 2014-12-10

**Authors:** Hiram Galeana-Zapién, César Torres-Huitzil, Javier Rubio-Loyola

**Affiliations:** Information Technology Laboratory, CINVESTAV-Tamaulipas, C.P. 87130 Ciudad Victoria, Tamaulipas, Mexico; E-Mails: ctorres@tamps.cinvestav.mx (C.T.-H.); jrubio@tamps.cinvestav.mx (J.R.-L.)

**Keywords:** context-awareness, energy-awareness, location-based services, middleware, mobile sensing applications

## Abstract

The disruptive innovation of smartphone technology has enabled the development of mobile sensing applications leveraged on specialized sensors embedded in the device. These novel mobile phone applications rely on advanced sensor information processes, which mainly involve raw data acquisition, feature extraction, data interpretation and transmission. However, the continuous accessing of sensing resources to acquire sensor data in smartphones is still very expensive in terms of energy, particularly due to the periodic use of power-intensive sensors, such as the Global Positioning System (GPS) receiver. The key underlying idea to design energy-efficient schemes is to control the duty cycle of the GPS receiver. However, adapting the sensing rate based on dynamic context changes through a flexible middleware has received little attention in the literature. In this paper, we propose a novel modular middleware architecture and runtime environment to directly interface with application programming interfaces (APIs) and embedded sensors in order to manage the duty cycle process based on energy and context aspects. The proposed solution has been implemented in the Android software stack. It allows continuous location tracking in a timely manner and in a transparent way to the user. It also enables the deployment of sensing policies to appropriately control the sampling rate based on both energy and perceived context. We validate the proposed solution taking into account a reference location-based service (LBS) architecture. A cloud-based storage service along with online mobility analysis tools have been used to store and access sensed data. Experimental measurements demonstrate the feasibility and efficiency of our middleware, in terms of energy and location resolution.

## 1. Introduction

The disruptive innovation of smartphones, provided with specialized sensors and more powerful computing and communication capabilities, is leading the development of cutting-edge mobile sensing applications applied to a broad range of sectors, like healthcare, social networks, transportation, among others [1]. It is expected that smartphones will also have on-board biometric, pressure and environmental sensors in the future, which will fully pave the way for smarter and more personalized applications. In any case, a common requirement of mobile phone sensing applications is that they need a client-side middleware application running on the smartphone for reading an internal phone's sensor and reporting sensed data to an external storage entity (*i.e.*, web server or mobile computing cloud) to enable either individual or community data analysis [1]. The data sensor acquired is typically sent over the wireless communication channel (e.g., *via* Wi-Fi or a cellular network) after locally performing a set of stages to select relevant features, filter redundant information and controlling data transmission behavior through the deployment and enforcement of low-level decision policies. At each stage, different algorithmic solutions have been envisaged to perform learning and classification tasks, and their main requirements depend on the application type and its impact on CPU and battery components. Innovations in battery technology have lagged behind the processing power of mobile devices, and thus, the design requirements of mobile sensing applications assume energy resources as the main constraint. As a matter of fact, according to McKinsey analysis [2], in the last decade, battery capacity has only doubled, while the processing speed of mobile phones has increased 12-fold.

In this context, energy resources are by far the most restrictive resource in smartphones nowadays. Furthermore, with the increased mobile phone usage in people's everyday activities, this trend is expected to persist in the future. The design and implementation of mobile sensing applications to reduce energy consumption and hence extend battery lifetime is of paramount importance in the mobile computing arena, especially to efficiently support applications that demand continuous sensing, that is highly frequent raw data acquisitions from sensors embedded in the smartphone [1]. In this regard, a commonly-used approach to save energy is to control the actions of sensors and suspend them when necessary. This is referred to in the literature as duty cycle adaptation, whose aim is to adjust parameters governing the sensor reading processes to reduce energy consumption. Notice that this approach is very attractive for mobile sensing applications that rely on location information, such as location-based services (LBSs), where the Global Positioning System (GPS), its core enabler, can significantly drain battery resources of smartphones if aggressive location readings are performed.

With this regard, the vast majority of previous works in the literature addressing the need of energy-efficient solutions have been focused on proposing design guidelines and strategies for energy optimization. In this sense, it is widely accepted that duty cycling of, for instance, a smartphone GPS receiver provides considerable energy efficiency (*i.e.*, increasing the sensing interval results in energy savings due to less use of the GPS receiver). As will be detailed later on in the paper, achieving such a goal for current smartphones is not straightforward. In particular, in an open source smartphone platform, like Android, the existing location application programming interfaces (APIs) only provide access to the system location services to obtain periodic updates of the device's geographical location. In the case of Android, the location manager does not allow on-the-fly sensing interval adjustments, which are crucial to efficiently control the duty cycle of the device and ultimately to reduce energy consumption.

On the other hand, we strongly believe that a middleware solution should provide a modular framework to deploy mechanisms to properly adjust sensing intervals without jeopardizing the quality of mobility analysis due to inappropriate resolution of location information at the GPS sensing stage. Note that sacrificing location resolution (*i.e.*, the poor resolution of GPS readings) might compromise the effectiveness and credibility of location-based information systems, which mainly rely on sensing location datasets. Therefore, a modular middleware design is needed to drive tradeoffs between energy consumption and location resolution for location-based applications (and more likely, for all sensor-based energy-expensive applications on smartphones).

In this context, in our previous work [3], we analyzed the main requirements for developing an energy-aware middleware for the support of location-based mobile applications with cloud computing interaction. In our attempts to fulfill the energy-efficient design requirements of mobile sensing applications, in this paper, we extend our previous work by proposing a novel middleware architecture that tracks the spatio-temporal evolution of users' positions to adapt the GPS sampling rate driven by user context-awareness in order to reduce energy consumption. The proposed middleware has been implemented between the application layer and the location manager in the Android software stack. It allows continuous location tracking in a timely manner and in a transparent way for the user. Basically, a service starts listening to location updates for some time interval and turns off after some elapsed time, removing all callbacks to the location manager. Then, the on and off times are re-scheduled dynamically depending on the way the user moves (e.g., speed, changes of speed, *etc.*), on energy resources availability and the target of the application that would enforce the context-aware energy savings approach proposed in this paper. In the following, we summarize the main contributions of our work:

- The key contribution of our work is the design of a novel mobile phone middleware architecture that provides support for the deployment of energy-efficient handling and transmission policies for sensor information streams via a novel lightweight API. Our middleware is highly modular. It is implemented on the Android platform, and it consists of three main building blocks: dynamic scheduler, mobility profiler and batch transmission. Each of these modules can be modified in isolation without affecting others, whereas high-level application-dependent sensing policies can be deployed and enforced easily in the middleware architecture.
- We highlight the existing research work done so far in the field and take a step forward by providing direct interfacing with the mobile phone' APIs and embedded sensors to enable effective duty cycle control based on observed context changes in the user situation. This latter idea is referred to as context-awareness, that is the dynamic adaptation of mobile phone sensing applications' configuration parameters according to the sensing surrounding context.
- We validate the proposed middleware solution assuming location as the key user context information considered in prominent sensing applications [4]. This information has been pivotal to developing predictive models of human behavior [5], which can be taken into account in other potential systems in the future. The adaptive location sensing mechanisms that control the duty cycle process to save energy are mainly based on location knowledge, and they are particularly tailored to fulfill the requirements of LBSs.
- Lastly, unlike most of the existing work in the literature, we integrate the proposed client-side middleware solution into a large-scale mobile phone sensing system that allows for continuous location tracking in a timely manner and in a transparent way for the user, maintaining a compromise with location resolution. As mentioned earlier, an LBS scenario is used to demonstrate the effectiveness of our middleware solution to efficiently collect sensor data and to fulfill data resolution requirements. However, our platform can be exploited in other applications that need to discover and take advantage of contextual information. We have conducted extensive experiments to evaluate the functionality of our middleware solution in Android smartphones using different test sets.

We strongly believe that the ideas presented in this work may encourage application developers to design energy-efficient applications in other application domains that require systemic sensing information from smartphones' sensors. The rest of the paper is organized as follows. Section 2 provides a summary of the related work. Then, we describe in Section 3 the current location sensing mechanisms in smartphones. Section 4 details the proposed middleware architecture for energy-efficient sampling and transmission of data sensor streams. In Section 5, we introduce a reference location-based mobility service framework in which the proposed solution is holistically validated. Section 6 presents experimental results to evaluate the benefits of our scheme. Then, mobility information quality is analyzed in Section 7, and finally, in Section 8, conclusions are drawn and future research directions are exposed.

## 2. Related Work

Location-based technology can be classified into two groups depending on the source of the signal to identify the location of users: terrestrial network-based methods based on various types of network measurements and satellite-based methods. The work presented in this paper falls into the second group, as the middleware exploits information acquired from GPS technology. Related work on the areas of this paper are provided hereafter.

Work by van der Spek *et al.* [6] developed a process and database architecture for collecting data on pedestrian movement, focusing on three European city centers. The authors made it evident that GPS offers a widely useable instrument to collect invaluable spatial-temporal data on different scales and in different settings, adding new layers of knowledge to urban studies. The authors highlight the use of GPS as a worldwide applicable sensor technique to collect spatial-temporal data and quantitative and qualitative information and its potential to carry out research in favor of urban planning and design. An application of the use of data acquired through location-based services is presented by van Lammeren *et al.* [7]. The authors present the Spatio-Temporal *in-situ* Experiences as Data (STEAD) approach for interpreting data to sense cultural-historic facts and anecdotes in landscapes, providing information about what has been sensed by whom, where and when, information that is exploited to define zones of interest. However, the critical aspects that have a direct impact on energy-efficient handling and transmission of sensor information streams is dismissed.

Different proposals for LBSs in the literature have taken into account energy constraints. For instance, in [8], a study on minimizing the power consumption of LBSs on mobile phones is presented. Different categories of LBSs are identified and grouped by service running time and power consumption. From a study of how existing LBSs consume power, the authors drew some conclusions: such services consume considerably more power than other mobile phone services; to save power, the GPS should be off as much as possible; and LBSs should try to reduce the amount of data transmitted to an external consumer. In relation to this, the authors in [9] identified four factors that lead to energy consumption in mobile devices: static use of the location sensing mechanism, absence of the use of other sensors, lack of cooperation among applications and ignoring the battery level while sensing. Then, they proposed a location-sensing framework to improve energy efficiency as design principles on Android-based smartphones. At a higher level of abstraction, in [10], the authors utilize the location-time history of the user along with the user's past velocity to adaptively turn on GPS only when the estimated uncertainty exceeds a predefined threshold. Additionally, the authors propose user movement as estimated by accelerometer signal processing to save energy for further usage.

The authors in [11] present the SenseLess application to perform energy-efficient mobile sensing. The proposed application makes use of less power-hungry sensors (e.g., accelerometer) as a means to augment location change detection in LBSs. SenseLess is able to detect when a user is not moving, and then, it stops sensing the GPS position to save energy. It makes use of a GPS sensor, and when no GPS signal is detected, it resorts to Wi-Fi technology to acquire location information. The authors argue that compared to a GPS-only approach, the SenseLess application is able to reduce energy consumption by more than 58% when determining the user's location and maintaining the accuracy of the sensed data. This approach, however, does not consider the resolution of locations, which is of paramount importance for LBSs, as it is directly related to the quality of information.

The work in [12] proposes a continuous sensing engine for smartphone applications that requires continuous monitoring of human activities and context. It develops different sensing, processing and classification pipelines for three smartphone sensors (accelerometer, microphone and GPS). Similar to [11], motion context change is detected using the accelerometer, and then the GPS sampling rate is adjusted, taking into account the combination of mobility detection, sensing duration and remaining energy budget. Additionally, it adapts the duty cycle when any of these factors change. Focusing on the GPS sampling mechanism, the authors in [12] propose an adaptive sampling schedule based on a Markov decision process that aims to estimate the GPS sampling schedule to minimize a given location error requirement. However, the applicability of this approach to other applications is not straightforward, as new pipelines need to be constructed according to specific requirements.

In [13], Global-Sense (G-Sense) is described as a middleware with an architecture that integrates wireless sensor networks, both static and mobile, with LBS support and monitoring of vital human signs. G-Sense includes mechanisms to reduce the amount of generated and transmitted data without compromising the application requirements. As an example, the GPS reading frequency can be adapted depending on the detection of user movement: if the user stops in a place, the frequency slows down; otherwise, it is increased to guarantee user tracking. G-Sense considers the computation and power constraints present in the wireless nodes and defines strategies to solve them.

Although it is clear that increasing the GPS sampling rate involves more samples per time unit and, thus, more energy consumption, adapting the sensing rate based on dynamic context changes through an efficient middleware architecture has received relatively little attention in the research literature. In this problem, it is very hard to efficiently and appropriately identify a context change that, in turn, could control (reduce/increase) the sampling rate. It is worth noting that, if by chance, it is determined that a user is not moving and then the GPS sampling rate is decreased, a poor location resolution would be achieved, and this would have a negative impact on the credibility of a given location-based service.

Based on previous research, the aims of this work are to provide: (1) a more integrated solution for on-device energy-awareness by designing a middleware solution to enable adaptive sampling and data transmission derived from user mobility contexts; and (2) a framework that goes beyond traditional LBSs that mostly provide personalized services by exploiting user's geographic location and average traveled distances or services limited to tracking the mobility of mobile entities.

## 3. Location Sensing Mechanisms in Smartphones

Today's smartphones support multiple location-sensing mechanisms, which make use of embedded GPS sensor information, as well as information from cellular networks (e.g., [14,15]) to get the assisting data from the Internet. For instance, in the Android OS, the location manager provides access to the system location services to obtain periodic updates of the device's geographical location through two different alternatives: GPS_PROVIDER and NETWORK_PROVIDER. In the former service, location is determined using satellites, whereas in the second case, location is retrieved based on availability of cell towers and Wi-Fi access points. In any case, it is important to note that the location manager approximation error can be quite large when using NETWORK_PROVIDER, and there is no guarantee that the user is inside the detected coordinates' round (and the round radius is less than the error). The reason is that the provider detects only the closest existing network node, but not the user's location. This also happens if the device connects to the Internet through Wi-Fi and it does not have a subscriber identity module (SIM) card on it. Additionally, GPS is the best technology for location accuracy; however, it has a drawback in that it could require up to two minutes to fix the position (*i.e.*, time to first fix) in non-appropriate signal conditions (for instance, near windows in an indoor environment). In any case, the provided accuracy of GPS (between 1–50 m) is quite acceptable in comparison with other alternatives: network-based (100–5000 m) and Wi-Fi (25–200 m) [16].

In addition, it is also possible to acquire location information through mobile operators' access networks using the location-based IP technology referred to as the Open Mobile Alliance (OMA) secure user plane location (SUPL) architecture [17]. SUPL has become extensively popular in recent years, because of its capability to use existing protocols and an opportunity for third party providers to support Assisted GPS (A-GPS), which combines satellite-based technology and ground-based approaches. It constitutes an alternative network and protocol architecture for mobile operators to promptly transfer location information throughout cellular networks and, therefore, to reduce the time response of the GPS technology. As mentioned above, SUPL is intended to be used along with A-GPS. Android OS already supports SUPL-specific mobile data connection in the connectivity manager (TYPE_MOBILE_SUPL).

In this work, we consider an LBS scenario that is based on GPS technology. We analyze the performance of our proposed middleware architecture in terms of the effective adaptation of sensor reading processes to achieve energy savings and the required location resolution. From experimental evaluations, we estimate the location error obtained from all collected datasets and validate the effectiveness of the middleware solution to deploy efficient sensing mechanisms to reduce energy costs in continuous location sensing applications.

## 4. Green Context-Aware Middleware

Despite the advances provided by hardware manufacturers and operating system vendors, continuous accessing of sensing resources in mobile devices to support location- and context-aware applications and services is still expensive in terms of energy [18]. Because of the potential benefits, embedding energy-awareness in the design, in the device and the protocols of the networks is highly desirable. Notice that although current location API methods in Android give access to the system location services, they do not support dynamic adaptation of sensing parameters on-the-fly. Recall that GPS handling facilities in Android are mainly applications working on the foreground that require few location updates, and the configuration parameters are set statically. Thus, this work offers an efficient solution to adapt sensing rate and energy-aware transmission within a novel green context-aware middleware. The middleware architecture has been designed to provide support to a wide range of LBSs using different location-sensing methods, like the ones presented in the previous section. In other words, our proposed middleware solution is not linked to a specific and unique location provider or to a specific technology to acquire location updates. This means that a smartphone running our proposed middleware solution can be configured to connect with a given location provider or mobile operator using a location-based IP technology, such as the OMA SUPL architecture [17].

**Figure 1.**
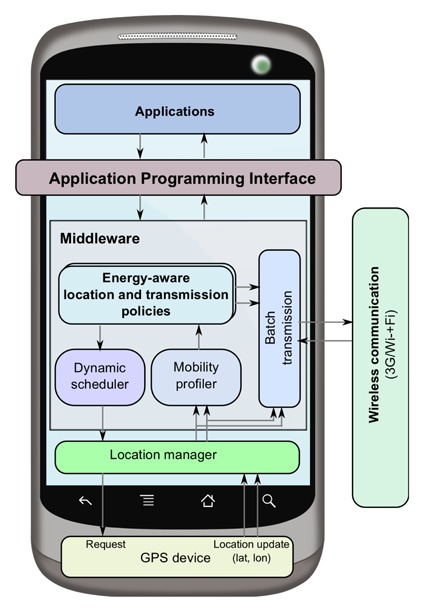
Middleware architecture for context-aware and energy-efficient sampling and transmission of data sensor streams.

Figure 1 shows the conceived middleware architecture for energy-efficient handling and transmission of sensor streams that provide a runtime environment for applications through an API [3]. The core components of the middleware are the dynamic scheduler, the mobility profiler and the batch transmission module that, in cooperation with the mobile operating system, interact with the mobile sensors for energy control purposes. These components are highly modular, so that component functionality can be modified in isolation without affecting the others. On top of this layer, high-level application-dependent policies for energy-aware location and transmission can be defined and inserted.

### 4.1. Dynamic Scheduler

The dynamic scheduler component of the middleware allows to simplify the sensor reading processes in the mobile device. In Section 4.5, we describe an implementation of the middleware on an Android operating system in an attempt to solve the problem of the lack of proper and flexible mechanisms that can limit the energy consumption due to uncontrolled location sensing Android applications that otherwise would retrieve GPS data continuously. Sensor access is simplified by providing a single software layer that enables agile development of mobile applications that require one to specify parameters to low-level energy-efficient rate-adaptive duty-cycling for GPS-based positioning, *i.e.*, dynamic adaptation of sensing parameters. The periodic sensing interval of the dynamic scheduler can be selected to be flexible based on the granularity and energy consumption requirements given by the location sensing application. Under different scenarios, this layer can be used as a low-level enabler for further energy savings, as developers are able to request controlled location updates according to established location resolution and energy trade-offs.

### 4.2. Mobility Profiler

For continuous location monitoring, periodic duty-cycling of GPS is not appropriate, as this embedded sensor could deplete the battery in a few hours [3]. Thus, an energy-efficient usage of GPS is required, taking advantage of individual mobility patterns. The mobility profiler's main goal is to estimate/predict the system state and user mobility from GPS data streams to dynamically schedule position updates to minimize power consumption with some tolerance in position accuracy. The context states, e.g., static or moving fast, can be used as a basis to define high-level policies that group together a number of techniques to further reduce energy consumption according to specific applications constraints and mobility scenarios. By knowing the user mobility state and transitions, GPS updates can be rescheduled at specific times and trigger location readings according to some policies; for instance, sampling at a coarse grain when the user is rather stationary and raising the sampling rate gradually according to the estimated user speed. The mobility profiler in our middleware provides good hints of user mobility from GPS logs, so as to adaptively change the location sampling rate according to a policy.

### 4.3. Batch Transmission

The batch transmission module is the key enabler of energy-awareness. It reduces the communication energy overheads introduced by the transmission of GPS data to a remote system through the wireless media. This module caches position fixes locally, and then, it selectively transmits packed subsets of GPS location streams driven by an adaptive duty cycle set by the mobility profiler and an energy budget. Moreover, this is a key element that allows finding trade-offs between the relevance of the GPS data and the energy cost of transmitting such information through wireless access.

### 4.4. Energy-Aware Policies

A policy might be seen as a set of coupled techniques that can define energy-efficient rate-adaptive GPS sampling and transmission batches according to user mobility context. Together, the energy-aware policies module and the mobility profiler decide the sensor sampling rate based on the energy budget, the mobility pattern and the purpose of the mobile application for which the context-aware energy saving approach is applied. The mobility profiler's main role is to estimate and predict typical mobility patterns from GPS data streams to determine a system state (static, moving fast, moving slow, *etc.*), which, in turn, is used by the policy-based modules to adapt dynamically the sensor sampling rate and the batch transmission. In this sense, other developers can build domain-specific location-based applications that use *ad hoc* policies relying on the facilities provided by the mobility profiler and dynamic scheduler service. The domain-specific knowledge to improve energy consumption can be linked to the middleware through the energy-aware policy-based modules.

**Figure 2.**
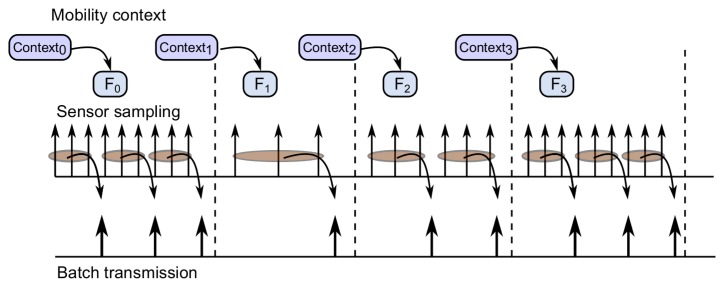
Conceptual view of the dynamic adaptation of sensing parameters and batch transmission.

Taking into account Figure 2, in the following, we explain the behavior of the key components of the middleware's functionality. The mobility profiler identifies the user mobility context, and it reschedules GPS invocations at specific times to enable the dynamic scheduler to adapt the interval sensing period. These features are particularly relevant in low mobility contexts (e.g., Context_1_), where significant energy savings can be achieved by increasing the time between two consecutive GPS invocations whenever possible. As will be shown later on in the paper, we validate the effectiveness of the dynamic scheduler in terms of energy savings by implementing three different policies; however, our middleware architecture is not restricted to these three energy-aware policies to control the adaptation of sensing parameters, as other policies can be configured to fulfill the requirements of the target application that would make use of the context-aware energy saving solution proposed in this paper.

The batch transmission of location data optimizes the use of energy resources in the device as a set of GPS samples that can be packed and transmitted through the wireless network. For instance, in Figure 2, a batch transmission size of three GPS readings is graphically shown. The integration of the aforementioned components results in different spacing for batch transmissions. The larger the sampling rate, the shorter the transmission spacing. All components of the location-based service framework, including the energy-efficient context-aware mobile application presented in this paper, have been designed and implemented as open source components. All of them can be made available to the interested readers and practitioners upon request.

### 4.5. Middleware Deployment on Android

The proposed middleware has been implemented in Android to enable the dynamic management of location resources and access control to assist continuous monitoring and long running proactive location-based services. In our proposal, the middleware provides a power management layer, in between the application layer and the location API layer in the Android software stack, as shown in Figure 1. Developers will request location updates through the middleware instead of requesting it directly from the underneath location API. Recall that the Android SDK provides a dedicated API for location information that is designed in a generic way, such that the same API can be used to retrieve location information from different localization techniques by using the location provider that uses the GPS receiver embedded in the device or the network facilities to get a location estimate. However, the Android built-in API is not intended for continuous location monitoring as a background service with a variable duty cycle. A well-known problem with near-constant-rate duty cycling is that it does not take into account the rate by which the user location actually changes or whether or not the user motion is uniform.

The rationale behind the proposed middleware for smartphones, particularly for Android devices, is as follows. The explicit power management strategy to maximally conserve energy employed in modern smartphones based on an aggressive sleeping policy to suspend the whole system after a short period of user inactivity, has led to a considerable burden on developers by delegating the responsibility to keep the system on to execute a time-sensitive task [19]. Furthermore, application developers are also expected to perform the power management of individual components, such as the GPS, to ensure that when a change in its operating state is needed or scheduled, the smartphone can be woken up, even if it is in a low-power suspend state.

The middleware provides the ability to dynamically register and unregister location updates and to adjust the duration between two consecutive location updates on the fly by taking advantage of Android system facilities. On the one hand, to support background services under the aggressive sleeping policy, the Linux kernel power manager in Android, along with hardware support, provides a high-level mechanism, called wakelock, for preventing a smartphone from fully suspending. See the online Android developer guide for other two additional mechanisms, suspend notifiers and hardware wakeups, to keep the system from suspending or to wake it up [19]. Wakelocks are special objects with two associated APIs calls, acquire, the smartphone cannot be suspended, release, to allow the OS to turn on with the sleeping policy [20]. Both API calls can be used after initialization by both kernel-space and user-space programs. On the other hand, timers and alarms in the Android framework are system mechanisms used for waking up the smartphone from the suspended state to support services that should run at fixed time intervals. In this sense, taking advantage of such systems facilities, the middleware is able to schedule and set an alarm with the user-specified time specification by using an initialization API for a location request. Internally, the middleware maintains the registered alarm time and executes a call back function after a software timer fires to handle the location update.

We therefore designed and implemented APIs in order for mobile sensing applications to utilize the services of the proposed adaptive sampling scheme. As the proposed middleware is implemented in Java, it would be easily ported to different systems. Basically, the middleware will expose two additional parameters that users can determine when requesting location updates: time to specify when to sense and the maximum time allowed for location acquisition. The latter works as follows. When a new sensing request from an application arrives, the middleware will push the request to the Android location layer to turn on the GPS device and wait for the response of the GPS. When location information is reported or the maximum acquisition time is reached, the layer will automatically unregister the listener to stop requesting the location and schedule another one according to the scheduling module. The policies module controls the interval between consecutive samples at runtime based on the predictability of the user's mobility by specifying the time for sensing, registering and implementing a listener interface with the adaptive sensing service encapsulated as a JAR (Java ARchive) file. Policies can be defined by users according to application requirements.

## 5. Reference Architecture of LBSs

**Figure 3.**
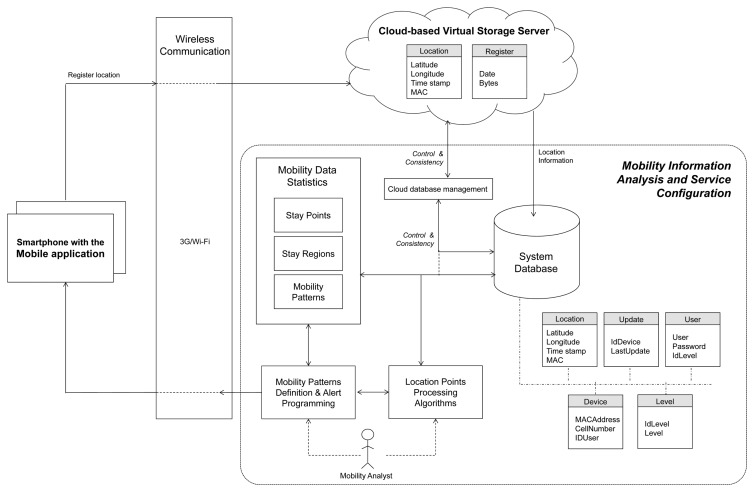
Location-based mobility service framework.

In this section, we describe a reference architecture to validate the main concepts developed in this paper. We describe a system architecture that allows one to transmit and store large amounts of sensed data for LBSs. This system architecture, referred to as the location-based mobility service framework, consists of three main parts, as is graphically shown in Figure 3: (1) mobile devices running the middleware for energy-efficient handling and transmission of sensor information; (2) a cloud-based virtual storage service; and (3) a mobility information analysis and service configuration part. The elements of the three parts of the reference architecture are described hereafter.

### 5.1. Mobile Devices

The mobile devices considered in this framework are smartphones that provide interfaces to take measurements from the embedded sensors, like GPS, proximity, accelerometer, *etc.* For instance, smartphones with the Android OS would support the development of mobile phone sensing applications; however, the framework is not restricted to such an operating system.

The green context-aware mobile application relies on a middleware architecture that enables the management of energy consumption based on low-level adaptive GPS reading and transmission schemes. GPS captures are sent to the cloud-based virtual storage server through the wireless access network infrastructure (3G/Wi-Fi). It is assumed that smartphones should have access to the Internet through a wireless operator network or through Wi-Fi access points. This way, the application can send the location data of the smartphone to the cloud-based virtual storage system on user mobility from anywhere.

### 5.2. Cloud-Based Virtual Storage Service

The cloud-based virtual storage server is a cloud database service in which location information is stored temporarily. The cloud service is adopted in our framework due to its flexibility to store data from different mobile sources, availability, lightweight requirements and practicality. However, a cloud-based approach is not widely recognized as the most appropriate alternative or as long-term storage due to privacy issues (*i.e.*, the collection of raw data information may compromise users' privacy). In this regard, access to the online database is granted by parameters deployed in the mobile application, namely the application makes use of the assigned port, the server IP address, and the authentication means (username and password) to access the database. A cloud-based storage is considered due to its potential to store a large-scale number of sensing applications concurrently to support ubiquitous individual and community context analysis for emerging LBS. It is worth noting that concerns about cloud storage, regarding reliability and security (see [21,22]), are out of the scope of this work.

The cloud database has a table to store the location information (identified as Location). It is used to store and to identify the latitude and longitude coordinates (expressed in sexagesimal degrees from −90 to +90 and from −180 to +180, respectively) from the mobile application, one running in each smartphone. It also stores the time at which the location points are acquired in the smartphone (in YYYY-MM-DD_HH:MM:SS format) and its MAC address. MAC addresses are used to appropriately identify the devices.

The cloud database includes also a table for capacity control. This is included in the system, because the total storage space of the database is a function of the capacity offered by the cloud service provider and also because storage resources are always limited. In order to control the storage capacity, a log of the date and the number of bytes sent by each smartphone's application are stored.

### 5.3. Mobility Information Analysis and Service Configuration

Mobility analysis tools are included in the reference architecture to evaluate the performance of our middleware solution to find trade-offs between energy savings and location resolution. Moreover, the improvement of the analysis tools presented herein is out of the scope of this work. This part of the framework allows us to configure mobility tools to be used in the analysis of the sensed information provided by the mobile devices. The mobility analysis framework includes a long-term storage system database where the overall mobility information is stored and maintained, as the cloud-based storage described earlier is used for temporary storage. We implement a control module to manage the amount and type of information stored in the cloud and the system database (cloud database management). This interface is used in our experiments to coordinate which and when information is downloaded to the system database for further use. The database downloads are carried out in three different time frames: (i) when the cloud-based storage has reached a threshold defined by the administrator; (ii) when it is necessary to analyze information of a specific user (or set of users) and the required information has not been downloaded to the system database; and (iii) when the periodic time to download has reached its limit. The preferable storage manager for the system database is MySQL, although the functionality of the framework is not restricted to it. The system database has five tables, as detailed in Table 1.

The above defined elements allow us to store and organize mobility patterns of the information provided by the mobile devices. Once sensed data have been collected and stored, we configure query conditions (the time and distance thresholds, and the start/end dates) for retrieving and processing location data. Specifically, in order to process the location information stored in the system database, we have implemented a searching strategy to mine GPS trajectories and to compute the following mobility statistics [23]. Location points are raw location coordinates collected from the mobile devices at a given day time. Stay points are location points that fall within time and distance thresholds. Stay regions are sets of stay points, which, in turn, represent regions of interest for the user of a mobile entity or users of a number of mobile entities.

We have included analysis algorithms to infer mobility patterns from the aforementioned mobility information. These algorithms are also used to detect stay regions of mobile users based on location information stored in the system database [23,24]. These algorithms analyze the trajectories of users and detect zones where users have remained for some time within some distance threshold. Furthermore, the algorithms extract additional statistics on the mobility behavior of mobile users, such as distance, average speed and traveling time.

We have selected stay point and stay region statistics, as they can be used further to develop sophisticated LBSs, like alert services to the mobile users. Alerts provide support, for example, in finding/receiving alternative paths in response to eventualities and contingencies that could be detected by the system as a result of the correlation of other users' mobility patterns. In this sense, alerts are the result of online information analysis of several mobile clients of the platform. It is worth noting that such a type of advanced location services can be deployed to exploit, for instance, the user's location and context-aware mobility patterns [25,26]. However, the development of specific LBSs (e.g., to proactively build alternative roads on maps due to emergencies, *etc.*) is out of the scope of this work.

The pivotal element of analysis in the above framework is the location information collected by the mobile application. The collection of such information from GPS sensors is energy expensive for the mobile entities. In order to improve energy efficiency, this work deals with the critical nature of regulating sensing intervals, as well as controlling the information upload streams in response to energy- and context-aware policies, as described earlier in the presentation of our middleware solution.

## 6. Performance Evaluation

In this section, we present the performance of the proposed middleware architecture implemented on Android smartphones and validated through experimental outdoor scenarios in Ciudad Victoria, Mexico. Experimental analysis aims to demonstrate the feasibility and efficiency of the dynamic scheduler, mobility profiler and batch transmission functionalities. We use the Samsung Galaxy III smartphone in all of the experiments. The testing mobile application, relying on the middleware, recorded the GPS logs, a time stamp and the battery information during the experiments that were collected and then pushed to the cloud-storage service. In the experiments, the smartphone was fully charged to counter the influence of the non-linear voltage decrease and turned on only the location sensor that we intended to measure. So as to analyze the power consumption profile, the battery level was recorded periodically and locally stored, and then, data were extracted for off-line analysis. In our experimental setup, we have enforced three possible sensing policies in our proposed green context-aware middleware solution to validate its effectiveness to efficiently provide support for handling and for the transmission of sensor streams. It is worth noting that a different type of sensing policy can be easily deployed in the middleware without the need to modify other modules, in cases when different behaviors of sensor reading and batch transmission schemes are needed. In this regard, we assume a delay-tolerant approach for location information processing at the mobility analysis service, and therefore, delay constraints are in this case neglected.

### 6.1. Fixed Duty Cycle Policy

In this scheme, the length of the sensing interval is fixed to a predefined value, so that GPS readings in each sensing cycle are performed regardless of the user's context. This unaware-context policy is used for benchmarking purposes, as we consider this as the worst-case possible situation in terms of energy consumption. In order to limit the cost associated with this continuous reading approach, we reduce the communication overhead by enabling batch transmissions.

### 6.2. Region-Based Policy

In this policy, the sensing interval that controls GPS invocations is increased when the context of a user is static (*i.e.*, the user does not move farther than a certain distance threshold with respect to its previous location). Note that increased sensing intervals are likely to result in more benefits in terms of energy savings, but the obtained data quality might be low for short-term stay point and stay region detection. On the other hand, remaining in a stationary state produces a reduction in the sensing interval up to the minimum sensing value. In practice, this approach leads to less frequent location updates and reduces energy costs, but resolution of locations cannot be guaranteed, especially if the context between two consecutive GPS sampling points changes. We thus aim to overcome this drawback in the third sensing policy discussed in the following.

### 6.3. Green Context-Aware Policy

This approach is intended to dynamically adapt sensing intervals based on context changes, while maintaining a minimum granularity of information. As to location resolution, we assume a reference distance to be satisfied between two consecutive sensor readings, and sensing intervals are adapted to the observed speeds. In this policy, the sensing adaptation process performed is also restricted within a minimum and a maximum sensing interval. In any case, context change is difficult to detect, especially after a long period in a stationary context. Note that in such a case, sensing intervals are likely to be adjusted to the maximum allowed value, which might result in location information loss if a transient context change takes place in the meantime. Moreover, the false context change detection problem makes the design of adaptive sensing mechanisms more challenging.

In this regard, the context change detection approach considered in our green context-aware policy is two-fold. Firstly, in order to avoid false context changes, *i.e.*, due to instantaneous speed values, an exponentially weighted moving average (EWMA) filter, namely a low pass filter, is applied to the observed speeds to smooth the resulting speed value. The EWMA filter is defined by Equation (1):

(1) $$\text{EWM}A_{i}=(1-\alpha )\text{EWM}A_{i-1}+\alpha V_{i}$$

where *I* ≥ 1,0 < *α* < 1. *EWMA_i_* is the current estimated EWMA value; *EW M A_i_*_−1_ is the previous estimated EWMA value; *V_i_* is the current speed of the mobile user; and *α* is the smooth filter factor. This approach constitutes a context-sensitive adaptation scheme, which is particularly useful in transitions from low to high-mobility scenarios. In this case, the adaptation of sensing intervals is strongly coupled with user speed changes, which are computed as follows. Given two location points obtained at two consecutive time instants, denoted as *L*(*i*) and *L*(*i −* 1), we firstly estimate the distance (in meters) between both points using the haversine formula:

(2) $$\alpha =\sin^{2}\left(\frac{\Delta \varphi }{2}\right)+\cos(\varphi_{1})\cdot \cos(\varphi_{2})\cdot \sin^{2}\left(\frac{\Delta \lambda }{2}\right)$$

where *φ* and *λ* are the latitude and longitude of a given location, respectively. Assuming that the radius of the Earth is given by *R*, the distance between the two consecutive points is obtained as:

(3) $$d_{L(i),L(i-1)}=R\cdot \left(2\cdot \text{atan}\cdot 2\left(\sqrt{a},\sqrt{\left(1-a\right)}\right)\right)$$

Along with the distance computation, we also obtain the elapsed time between the two GPS points by subtracting the corresponding time stamps. Finally, with distance and time values, the mobile user speed can be estimated.

On the other hand, unnecessary reductions of the sensing intervals are likely to happen due to eventual context change false detection (*i.e.*, inferring a change from a non-stationary state to a stationary state). In this case, we track sensing interval adjustments performed by the dynamic scheduler during a reference sampling window (*i.e.*, a number of consecutive GPS samples). Taking this into account, we again consider the EWMA value of sensing intervals and use it to upgrade the sensing intervals.

### 6.4. Experimental Results

**Figure 4.**
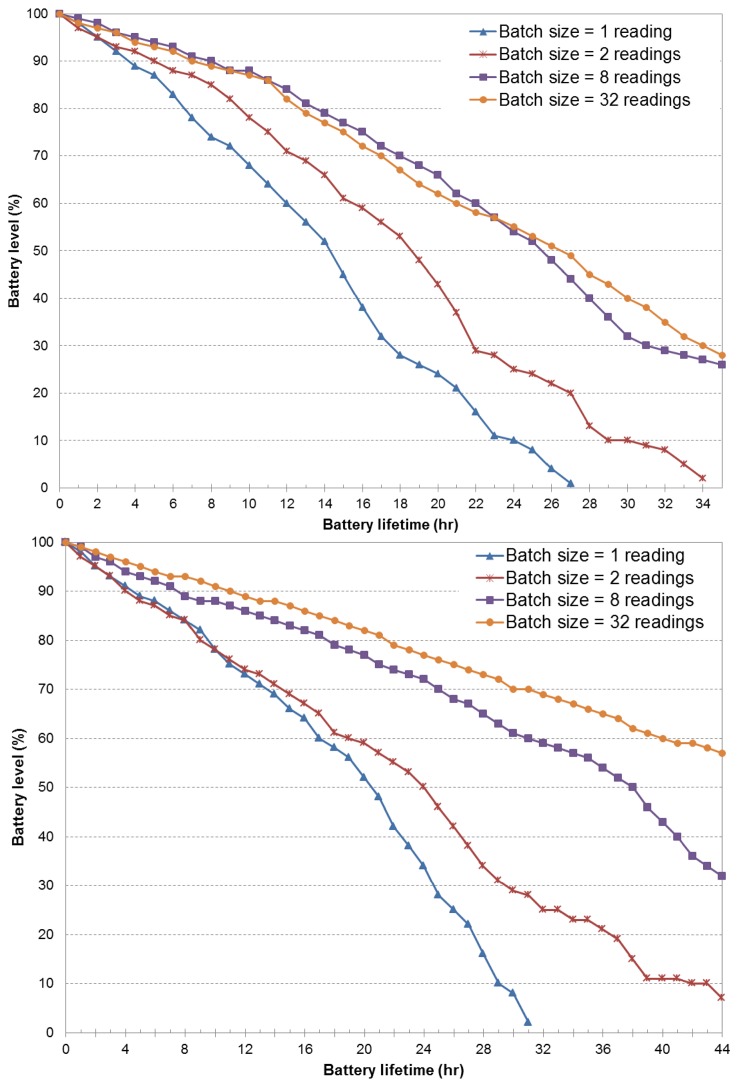
Battery lifetime of four batch transmission schemes under different fixed sensing intervals: (**Top**) two minutes; (**Bottom**) four minutes.

Figure 4 presents the battery level *versus* the battery lifetime obtained as a result of the use of the fixed duty cycle policy. We assumed fixed sensing intervals of two and four minutes, and four batch transmission sizes: 1, 2, 8 and 32 GPS readings. Figure 4 (top) demonstrates that the poor performance of single transmissions can be enhanced by allowing batch transmission mode. Individual transmissions of sensor samples demands around 40% of battery resources to achieve a battery lifetime of 12 h. For the same lifetime value, batch transmission sizes of 32 GPS readings consume only 15% of battery resources. Packing and transmitting the GPS data in batches or bursts allow the wireless radios to operate on a low duty cycle to better amortize the energy overheads of wireless transmissions as a result of lower bandwidth usage and a reduction of data traffic.

Furthermore, combined batch transmissions and increased sensing intervals (e.g., 4 min), extend the battery lifetime. As shown in Figure 4 (bottom), the battery drain reduction is very noticeable, even for the case of individual transmissions. By comparing both figures for a battery level of 80%, the sensing interval of four minutes and batch transmission of 32 samples are able to achieve a higher battery lifetime (20 h) than the case of a fixed two-minute sensing interval (13 h). The major portion of energy savings is assessed due to a more efficient header-to-payload ratio. Intuitively, energy consumption of data transmission in mobile (radio) devices is highly dependable on the traffic pattern, that is the larger the amount of sensing information transmitted over the wireless channel, the larger the energy cost observed in the experiments. In any case, the batch transmission scheme is intended to limit the cost of continuous sensing and to reduce the communication energy cost. This is achieved by packing sensor data instead of sending all sensed (raw) data information over the wireless interface. However, as larger batch transmission sizes are considered, transmission schemes tend to behave similarly (e.g., eight and 32 GPS readings), implying that both approaches result in a similar header-to-payload ratio. Notice that in both eight and 32 batch transmission experiments, the sampling rate behaves very close to the maximum time for a GPS to lock, which, in turn, results in a GPS sensor being on most of the time.

**Figure 5.**
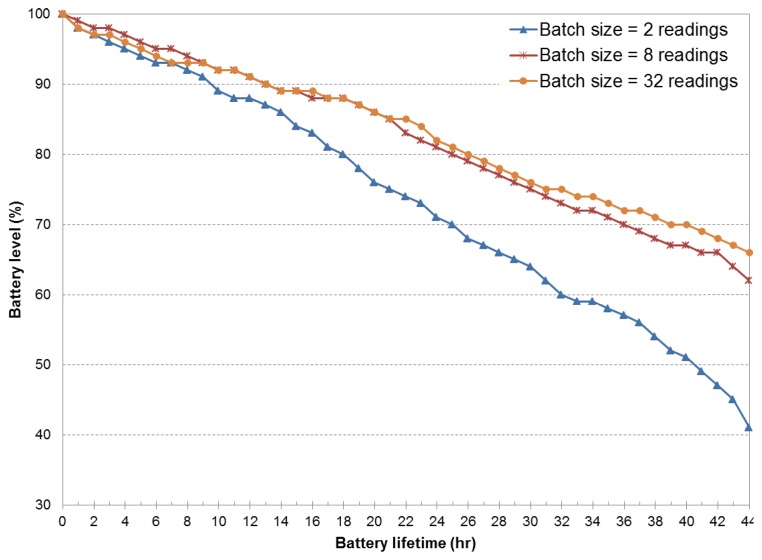
Battery lifetime of region-based sensing policy with three batch transmission schemes and variable readings.

In the next set of experiments, we evaluate the performance of the batch transmission functionality within the middleware's dynamic scheduler along with the defined region-based scheduling policy. Results are presented in Figure 5. We observe that small batch sizes take more advantage of this region-based notion. This comes to the fact that sensing intervals are increased as a result of low distance displacements. That is, the sensing interval is linearly increased (assuming steps of 2 min) when the distance between the current GPS sample and the previous one is equal to or lower than 100 m. The minimum and maximum allowed sensing interval is set to 2 and 16 min, respectively. It is clear that the duty cycle obtained by increasing the sensing interval brings benefits to battery resources. However, it disregards location information granularity, which is highly desirable for enhanced analysis of location-based services.

**Figure 6.**
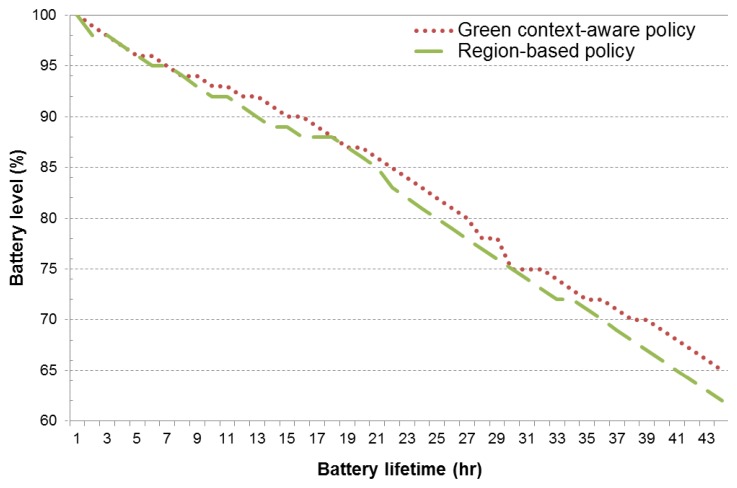
Battery lifetime of green context-aware sensing policy with a batch size of eight samples and variable reading intervals.

In the following, we evaluate the green-context aware sensing policy and compare it to the region-based approach. In both cases, a batch transmission of eight samples is considered, along with the aforementioned sensing interval thresholds. From the obtained results (see Figure 6), we observe that the proposed policy is able to achieve a 3% battery energy savings (for a lifetime of 44 h) over the region-based approach. It is worth noting that the green context-aware policy achieves this performance improvement by exploiting a relatively small period of time, where the user is in high-mobility scenarios (*i.e.*, around 5% to 10% of the total time).

**Figure 7.**
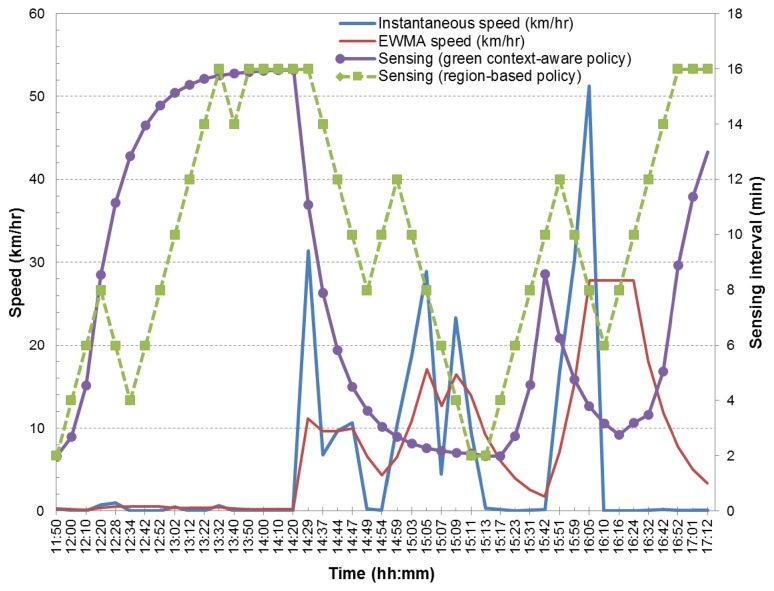
Behavior of adaptive sensing intervals based on observed speeds.

An illustrative example of the above is given in Figure 7, and we evaluate the responsiveness of adaptive reading to context changes by analyzing sensing interval adjustments over a period of time of nearly six hours. This is illustrated in Figure 7 for two different sensor reading and transmission conditions, namely through the use of the region-based policy and the green context-aware policy. The figure also shows the instantaneous and EWMA speed values (with *α* = 0.125 in Equation (1)). It can be seen that when context changes are frequently subject to user's mobility behavior, the green context-aware policy leads to smooth transitions between minimum and maximum sensing interval thresholds. On the other hand, the region-based policy leads to frequent sensing intervals corrections as a result of the bad behavior of its context change detection. Furthermore, false context change detection is prevented in the green context-aware policy by adjusting sensing intervals, taking into account a window history of three GPS samples.

## 7. Mobility Data Analysis

**Figure 8.**
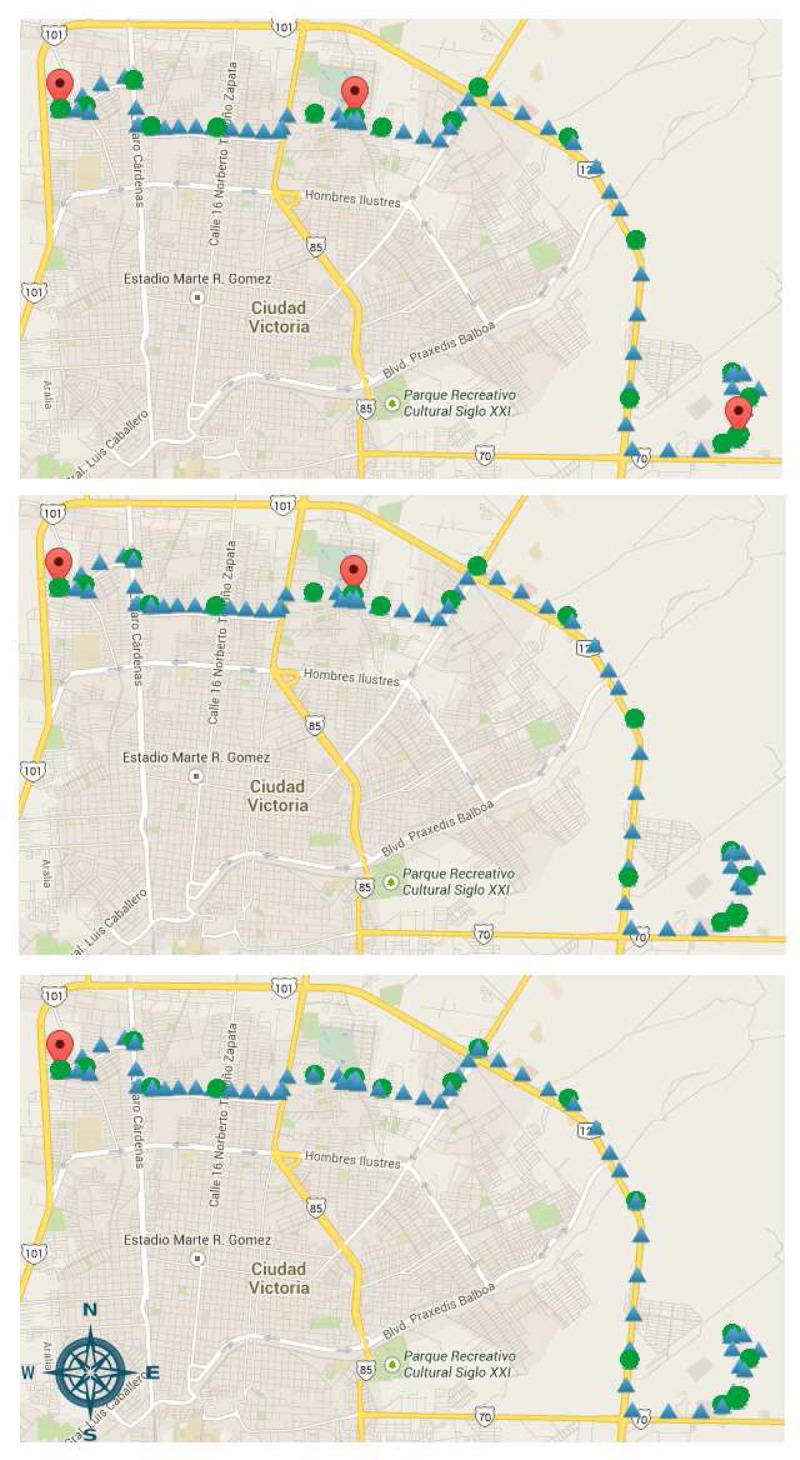
Stay region detection using the location point processing algorithm under temporal thresholds of 5, 10 and 15 min (from top to bottom) and a spatial threshold (three cases) of 100 m.

In this section, we perform the analysis of the spatial and temporal evolution of mobility trajectories through the use of the reference mobility analysis framework. Analysis of mobility data is performed by means of sensor data extraction from the cloud virtual storage and/or the system database. Figure 8 shows the result of location data extraction for different query predicates. For the illustrated trajectory, query processing is executed to compute stay regions assuming three stay time thresholds (5, 10 and 15 min) and a distance threshold of 100 m. Figure 8 shows the mobility trajectory followed by the user carrying a smartphone executing the developed mobile sensing application (green circles). As for mobile entities, we also record location readings obtained by a GPS logger device (blue triangles), able to continuously sample GPS location at a rate of 10 Hz. Both devices were charged by the same user at the same time during all experiments in the covered distance (10.46 km). For the sake of clarity, only a few samples are shown in Figure 8, as the logger was merely used to generate the reference trajectory, but the complete collected data might be used further for a more thorough assessment of the precision of the energy-aware policies. Given the GPS trajectory dataset, collected from the smartphone, stay points are firstly obtained to have a more meaningful representation of locations of a user's stay points. Then, by clustering the stay points, we obtain the stay regions (red location icons), which in practice can be used as inputs to determine, for instance, location-activity correlations. In particular, to discover places of interest in everyday life from users' location data (referred to as mobility data statistics in Figure 3), we use the algorithm proposed in [27]. We also observe that precise resolution of location records is directly related to the higher quality of context and mobility data analysis.

Finally, we estimate the average location error (ALE) of the GPS dataset collected from the smartphone. Formally, let *L_smartphone_*(*k*) and *L_logger_*(*k*) be the location reported by the smartphone and the location provided by the GPS logger device, respectively, at a given point *k*. Assuming *T* discrete time-GPS points, the average location error can be easily computed as [28]:

(4) $$\text{ALE}=\sum_{k=1}^{T}\left(\frac{L_{\text{smartphone}}(k)-L_{\text{logger}}(k)}{T}\right)$$

Following this approach, we estimate that the ALE is in the order of 20 m in the set of experiments performed. As a step forward, it is possible to formulate an optimization problem to find the GPS invocations that should be scheduled, such that the resulting ALE is minimized for a given energy budget. This type of experiment, however, is out of the scope of this work and will be part of our future work.

## 8. Concluding Remarks and Future Work

This article has proposed a novel middleware framework for embedding context-awareness functionalities to manage dynamic adaptation of sensing parameters and transmissions. The proposed solution is intended to improve the battery lifetime and to maintain an accurate data information granularity for energy-efficient location-based services. The solution builds upon the introduction of three modular components: dynamic scheduler, mobility profiler and batch transmission. The underlying mechanisms encompassing the designed architectural modules have been detailed. On such a basis, high-level application-dependent policies for energy-aware location and transmission have been implemented and validated through extensive measurements. Experimental results demonstrate the effectiveness of our mobile phone middleware in terms of battery lifetime due to a combination of batch transmissions and context-aware reading intervals. Furthermore, the proposed sensing approach is able to find trade-offs between energy savings and location data granularity. To gain insight into this issue, mining of location data is performed to highlight the impact of time thresholds on stay point and stay region detection capabilities.

We believe that context-awareness properties, together with location information, can fully pave the way to advanced sensing applications aimed at understanding both the individual and community behaviors in terms of mobility patterns and daily habits. Thus, as future research directions, we identify context prediction and context refinement based on an underlying context and energy awareness location-based service. Finally, due to the extensible design of the proposed middleware, we also aim to integrate multiple sensor reading processes, such as the accelerometer, in order to, for instance, detect when a pedestrian user is not moving, and then stop the GPS reading process, as localization in such cases is unnecessary.

## Figures and Tables

**Table 1.** Tables in the system database.

**Name**	**Description**
Location	Synchronized with the Location table in the cloud server. It also stores the location ID, location updates (latitude, longitude), time stamp and MAC of the mobile devices.
Device	Stores information of the mobile devices registered in the system: device ID, user ID, MAC address and the corresponding cell phone number to identify each mobile device.
User	Stores data related to both the mobile entities' users and the user of the analysis and configuration system (analyst).
Update	This table stores details concerning the last time and date when the location information of a certain device was updated in the system database.
Level	This table stores details of the access rights to the system database for analysts.
